# Predictive role of perioperative neutrophil to lymphocyte ratio in pediatric congenital heart disease associated with pulmonary arterial hypertension

**DOI:** 10.1186/s12893-020-01009-x

**Published:** 2021-01-04

**Authors:** Xiaoqiang Yin, Mei Xin, Sheng Ding, Feng Gao, Fan Wu, Jian Wang, Jie Chen, Li Jiang, Xiaochen Wu, Xianying Wang, Jingzhen Liu, Jinbao Zhang, Siyi He

**Affiliations:** 1 Department of Cardiovascular Surgery, Jinniu District, General Hospital of Western Theater Command, Rongdu Avenue No.270, Chengdu, 610083 Sichuan China; 2 North Sichuan Medical College, Nanchong, Sichuan China

**Keywords:** Neutrophil to lymphocyte ratio, Congenital heart disease associated with pulmonary arterial hypertension, Children, Cardiac surgery, Correlation

## Abstract

**Background:**

We aimed to explore the relationship between the neutrophil to lymphocyte ratio (NLR) and the early clinical outcomes in children with congenital heart disease (CHD) associated with pulmonary arterial hypertension (PAH) after cardiac surgery.

**Methods:**

A retrospective observational study involving 190 children from January 2013 to August 2019 was conducted. Perioperative clinical and biochemical data were collected.

**Results:**

We found that pre-operative NLR was significantly correlated with AST, STB, CR and UA (P < 0.05), while post-operative NLR was significantly correlated with ALT, AST, BUN (P < 0.05). Increased post-operative neutrophil count and NLR as well as decreased lymphocyte count could be observed after cardiac surgery (P < 0.05). Level of pre-operative NLR was significantly correlated with mechanical ventilation time, ICU stay time and total length of stay (P < 0.05), while level of post-operative NLR was only significantly correlated to the first two (P < 0.05). By using ROC curve analysis, relevant areas under the curve for predicting prolonged mechanical ventilation time beyond 24 h, 48 h and 72 h by NLR were statistically significant (P < 0.05).

**Conclusion:**

For patients with CHD-PAH, NLR was closely related to early post-operative complications and clinical outcomes, and could act as a novel marker to predict the occurrence of prolonged mechanical ventilation.

## Background

Congenital heart disease (CHD) associated with pulmonary arterial hypertension (PAH) refers to the increase of pulmonary artery pressure caused by CHD with shunt from systemic circulation to pulmonary circulation. PAH can occur at various stages in the evolution of CHD, and the degree of pulmonary vascular disease is regarded as a key factor affecting the efficacy and timing of surgery. Most CHD operations need to be completed at an early age. However, multiple organs are not fully developed in childhood, thus leading to high mortality and poor clinical prognosis. In addition, the inflammatory reaction induced by cardiopulmonary bypass (CPB) will influence the post-operative pulmonary and cardiovascular function and reduce the long-term survival rate of patients. It is reported that the systemic inflammatory response syndrome after CPB in children resulted in prolonged mechanical ventilation time, ICU stay time as well as total hospitalization duration [1]. Therefore, for patients with CHD, it is of great significance to explore effective clinical indicators in the early stage after CPB cardiac surgery to evaluate the level of inflammatory response.

Recently, a variety of studies have shown that inflammatory markers are closely related to the clinical outcomes of many cardiovascular diseases [2–4]. The neutrophil to lymphocyte ratio (NLR) has been paid more attention on due to its significant features of low price, immediate access and repeatable detection. Numerous evidences suggest that NLR is a novel marker which can reflect the level of systematic inflammation. As for cardiovascular events, NLR is also a remarkable predictive index. Recent studies perceived that the higher pre-operative NLR in children with CHD was not only associated with a higher risk for low cardiac output in the early post-operative period but also correlated with mortality [5–7]. Besides, Xu et al. found that post-operative NLR in pediatric patients with CHD after CPB was significantly higher than those before operation, which was consistent with increased mechanical ventilation time and intensive care unit (ICU) stay time [8].

Although NLR is a valuable indicator for inflammation, there is limited evidence in the literature of children with CHD-PAH. Therefore, the present research intends (1) to explore the correlation between NLR levels and early clinical outcomes in children with CHD-PAH undergoing CPB open heart surgery and (2) to evaluate the reliability of NLR level as a predictor for prolongation of mechanical ventilation time, thereby providing underlying predictor for clinical prognosis.

## Methods

### Subjects

This was a retrospective observational study conducted in the Department of Cardiac surgery of General Hospital of Western Theater Command from January 2013 to August 2019. Inclusion criteria: (a) consecutive patients less than 14 years old in our department; (b) according to 2015 guidelines by the European Society of Cardiology (ESC) and the European Respiratory Society (ERS) [9], the estimation of pulmonary arterial systolic pressure (PASP) is based on the peak tricuspid regurgitation velocity and right atrial pressure by echocardiography, and all patients were diagnosed with PAH when PASP was greater than 40 mmHg; (c) open heart surgery with CPB was performed. Exclusion criteria: (a) redo-surgery; (b) patient was diagnosed with any type of infection by clinical symptoms, signs and related examinations after admission; (c) patients received antibiotics therapy before operation; (d) patients received endotracheal intubation before surgery. The study was reviewed and approved by the Ethics Committee of General Hospital of Western Theater Command (No. 2020ky003). The informed consent form was given and signed by all guardians of the children. All methods were carried out in accordance with the ethical standards in the Declaration of Helsinki.

### Data collection

Perioperative clinical and biochemical data were collected retrospectively through the clinical electronic medical record system of our hospital. The clinical data included patient demographics (gender, age, weight, diagnosis), PASP, the Risk Adjustment in Congenital Heart Surgery version 1 (RACHS-1) score, CPB time, aortic clamping time, operation time, and follow-up clinical outcomes after surgical procedure including mechanical ventilation time, ICU stay time and total length of stay. On the morning of the first day after admission, peripheral venous blood was taken and sent to the laboratory department of our hospital for blood routine examination, and pre-operative NLR was calculated. After CPB cardiac surgery, peripheral venous blood was taken from the patients in the early morning of the first day in the ICU and sent to the laboratory of our hospital. The biochemical data included blood routine related indexes (neutrophil count, lymphocyte count and the calculated post-operative NLR), liver function related indicators (glutamic pyruvic transaminase, glutamic oxaloacetic transaminase, albumin and total bilirubin), renal function related indicators (creatinine, urea nitrogen, uric acid). The LVEF recorded by echocardiography was collected before discharge.

### Statistical analysis

All data were analyzed by SPSS25. Kolmogorov Smirnov test was used to evaluate the normal distribution of variables. The measurement data that approximate or conform to the normal distribution were expressed as mean ± standard deviation, while those that do not conform to normal distribution were expressed as median (quartile interval). The enumeration data were expressed as the corresponding number of cases. Two independent samples Mann–Whitney U test was used for comparison between groups. Spearman correlation analysis was used to evaluate the correlation between NLR and various Indexes. ROC curves were used to examine the various cutoff values of pre-operative and post-operative NLR to predict the need for mechanical ventilation beyond 24 h, 48 h and 72 h respectively. All tests were performed 2-tailed, with P < 0.05 considered statistically significant.

## Results

### Baseline characteristics

A total of 190 patients experienced CPB, including 104 males (54.7%) and 86 females (45.3%). The median age was 18.383 (7.367–49.367) months, and the median weight was 9.25 (6.5–15) kg. The median pre-operative PASP was 75 (67.75–82) mmHg. 135 patients were diagnosed as simple CHD, while 55 patients were diagnosed as complex CHD, which were minutely described in Additional file 1: Table S1. The results of RACHS-1 score was as follows: RACHS-1 (n = 12), RACHS-2 (n = 131), RACHS-3 (n = 44), RACHS-4 (n = 1) and RACHS-5 (n = 2). All patients received mechanical ventilation for 22.4 (5.917–47.596) hours, stayed in ICU after surgery for 2.856 (0.917- 5.864) days, and had a total length of stay for 18 (14–24.25) days. Detailed information was shown in Table 1.

**Table 1:** Baseline characteristics of included patients

Variable	Results
Pre-operative	
Male/female	104/86
Age (months)	18.383 (7.367–49.367)
Weight (kg)	9.250 (6.500–15.000)
PASP (mmHg)	75.000 (67.750–82.000)
Diagnosis	
Simple CHD	78
Complex CHD	112
RACHS-1 score	
1	12
2	131
3	44
4	1
5	2
Pre-operative neutrophil (10^9^/L)	2.595 (1.998–3.553)
Pre-operative lymphocyte (10^9^/L)	4.625 (3.008–5.848)
Pre-operative NLR	0.586 (0.404–1.022)
Intra-operative	
CPB time (min)	61.000 (52.000–72.000)
Aortic clamping time (min)	34.000 (26.750–47.000)
Operation time (min)	140.000 (130.000–160.000)
Post-operative	
Post-operative neutrophil (10^9^/L)	9.660 (7.860–12.190)
Post-operative lymphocyte (10^9^/L)	1.490 (1.090–1.873)
Post-operative NLR	6.499 (4.897–9.114)
ALT (U/L)	20.550 (17.400–26.800)
AST (U/L)	105.400 (80.625–133.85)
ALB (g/L)	42.203 ± 3.714
STB (µmol/L)	21.370 (15.868–30.125)
CR (µmol/L)	28.800 (23.000–35.000)
BUN (mmol/L)	6.690 (5.365–7.903)
UA (µmol/L)	312.650 (251.075–419.025)
LVEF (%)	60.000 (58.000–62.000)
Mechanical ventilation time (h)	22.400 (5.917–47.596)
ICU stay time (days)	2.856 (0.917–5.864)
Total length of stay (days)	18.000 (14.000–24.250)

PASP: pulmonary arterial systolic pressure; CHD: congenital heart disease; RACHS-1: risk Adjustment for Congenital Heart Surgery-1;NLR: neutrophil to lymphocyte ratio; CPB: cardiopulmonary bypass;ALT: glutamic pyruvic transaminase; AST: glutamic oxaloacetic transaminase; ALB: albumin; STB: Serum total bilirubin; CR: creatinine; BUN: blood urea nitrogen; UA: uric acid; LVEF: Left ventricular ejection fraction; ICU: intensive care unit

### Correlation between NLR and perioperative characteristics

First of all, we analyzed the correlation between pre-operative NLR and perioperative indexes of cardiac surgery. As shown in Table 2, pre-operative NLR was significantly correlated with AST, STB, CR and UA after operation (P < 0.05). However, there was no significant relationship between pre-operative NLR and PASP (P = 0.134), CPB time (P = 0.578), aortic clamping time (P = 0.547), operation time (P = 0.572), post-operative NLR (P = 0.212), ALT (P = 0.558), ALB (P = 0.14), BUN (P = 0.194) and LVEF (P = 0.268).

**Table 2:** Correlation between pre-operative NLR and perioperative indexes

Variable	Coefficient	P-value
PASP (mmHg)	− 0.109	0.134
CPB time (min)	− 0.040	0.578
Cross-clamping time (min)	0.044	0.547
Operation time (min)	0.041	0.572
Post-operative NLR	− 0.091	0.212
ALT (U/L)	− 0.043	0.558
AST (U/L)	− 0.252	0.000**
ALB (g/L)	− 0.107	0.140
STB (umol/L)	− 0.242	0.001**
CR (umol/L)	0.166	0.022*
BUN (mmpl/L)	− 0.095	0.194
UA (umol/L)	− 0.174	0.016*
LVEF (%)	0.081	0.268

* Indicates significant correlation at level 0.05

** Indicates significant correlation at level 0.01

Secondly, we explored the pertinence between post-operative NLR and liver function, renal function and LVEF after cardiac surgery. The results revealed that post-operative NLR was significantly correlated with ALT, AST and BUN after operation (P < 0.05) (Table 3). No significant correlation could be observed between post-operative NLR and post-operative neutrophil count (P = 0.054), post-operative lymphocyte count (P = 0.224), ALB (P = 0.183), STB (P = 0.125), CR (P = 0.087), UA (P = 0.162), LVEF (P = 0.605).

**Table 3:** Correlation analysis of post-operative NLR with post-operative indicators

Variable	Coefficient	P-value
Post-operative neutrophil (10^9^/L)	− 0.140	0.054
Post-operative lymphocyte (10^9^/L)	− 0.089	0.224
ALT (U/L)	0.213	0.003**
AST (U/L)	0.165	0.023*
ALB (g/L)	0.097	0.183
STB (umol/L)	0.112	0.125
CR (umol/L)	− 0.125	0.087
BUN (mmpl/L)	0.264	0.000**
UA (umol/L)	0.102	0.162
LVEF (%)	0.038	0.605

* Indicates significant correlation at level 0.05

** Indicates significant correlation at level 0.01

### Comparison of perioperative neutrophil count, lymphocyte count and NLR

Due to inflammatory responses caused by CPB, post-operative neutrophil count at the 1st day after surgery was significantly increased compared with pre-operative corresponding level (P < 0.05), while the post-operative lymphocyte count was significantly decreased (P < 0.05). The median pre-operative NLR was 0.586 (0.404—1.022), which markedly increased to 6.499 (4.897- 9.114) post-operatively (P < 0.05) (Table 4).

**Table 4:** Comparison of perioperative neutrophil count, lymphocyte count and NLR

Variable	Pre-operation	Post-operation	P-value
Neutrophil	2.595 (1.998–3.553)	9.660 (7.860–12.190)	0.000**
Lymphocyte	4.625 (3.008–5.848)	1.490 (1.090–1.873)	0.000**
NLR	0.586 (0.404–1.022)	6.499 (4.897–9.114)	0.000**

** Indicates rank sum test at level *0.01*

### Correlation between NLR and clinical outcomes

As presented in Table 5, pre-operative NLR was significantly correlated with mechanical ventilation time, ICU stay time and total length of stay (P < 0.05). Post-operative NLR was closely related to mechanical ventilation time and ICU stay time (P < 0.05), but had no obvious correlation with total length of stay (P = 0.161).

**Table 5:** Correlation between NLR and clinical outcomes

Variable	Pre-operative NLR		Post-operative NLR	
	Coefficient	P-value	Coefficient	P-value
Mechanical ventilation time	− 0.156	0.032*	0.361	0.000**
ICU stay time	− 0.227	0.002**	0.168	0.020*
Total length of stay	− 0.171	0.018*	0.102	0.161

* Indicates significant correlation at level *0.05*

** Indicates significant correlation at level *0.01*

### Predictive value of NLR for prolonged mechanical ventilation

**Fig. 1:**
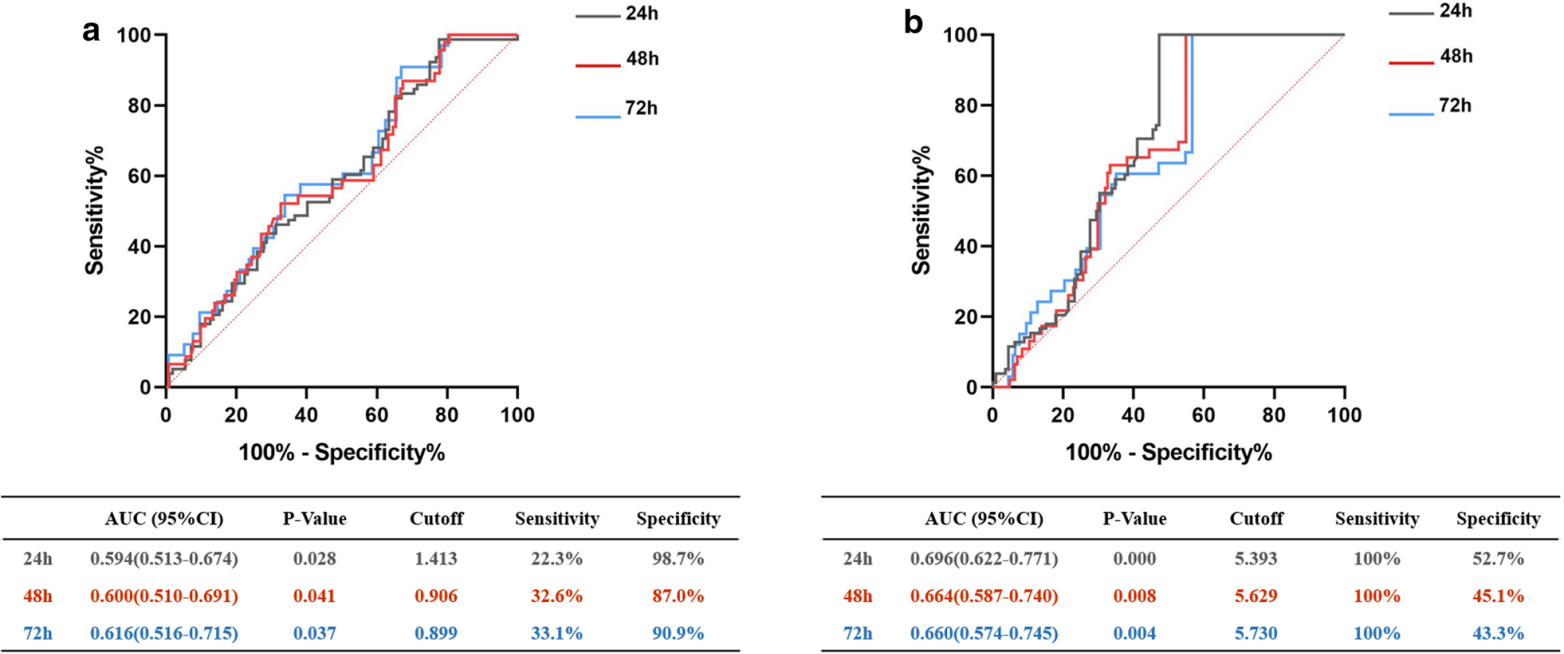
Predictive value of NLR for prolonged mechanical ventilation. **a** ROC curve for pre-operative NLR predicting the need for mechanical ventilation time beyond 24 h, 48 h and 72 h. **b** ROC curve for post-operative NLR at the first day after CPB cardiac surgery predicting the need for mechanical ventilation time beyond 24 h, 48 h and 72 h. AUC, Area under the curve; CI, confidence interval

At first, pre-operative NLR was studied for the value in predicting the prolongation of mechanical ventilation. The corresponding sensitivity and specificity of pre-operative NLR predicting mechanical ventilation time beyond 24 h, 48 h and 72 h respectively, as well as the best cutting point of pre-operative NLR, were shown in Fig. 1a. Subsequently, we analyzed the reliability of post-operative NLR in predicting the prolongation of mechanical ventilation time. The relevant sensitivity and specificity of post-operative NLR predicting mechanical ventilation time over 24 h, 48 h and 72 h respectively, as well as the best cutting point of post-operative NLR, were demonstrated in Fig. 1b. The areas under the ROC curve of NLR predicting mechanical ventilation time extension 24 h, 48 h and 72 h were statistically significant (P < 0.05).

## Discussion

CHD-PAH belongs to the first category in the classification of pulmonary hypertension and is a common complication of CHD with left to right shunt. Once it occurs, it will greatly increase the risk for CHD surgery. Therefore, early surgical repair of defects is deemed as a significant way to correct PAH thoroughly. However, the systemic inflammatory response caused by CPB with open heart surgery seemed to associate with high frequency of post-operative neuromotor disabilities [10]. A prospective cohort study suggested that neutrophil phenotypic was regarded to be correlated with post-operative acute kidney injury in infants undergoing CPB and could be used to predict inflammatory organ dysfunction [11]. The results of our study also confirmed that the level of inflammation in children with CHD-PAH significantly increased after CPB open heart surgery.

Recently, many studies have revealed the role of hematological parameters in predicting the prognosis of patients with CHD, including BNP, N-terminal pro-brain natriuretic peptide (NT-proBNP) [12, 13], serum troponin [14], C-reactive protein (CRP) [15], Red blood cell distribution width (RDW) [16], NLR and so on. NLR reflects not only the balance between neutrophil count and lymphocyte count but also the degree of systemic inflammation, and is related to the severity degree of cardiovascular disease. Previous studies have found that NLR was considered to predict the worsening of the renal function in diabetic patients [17]. In a prospective multicenter cohort study, Ackland et al. suggested that pre-operative NLR greater than 4 was associated with perioperative myocardial injury and systemic inflammation might contribute to the development of perioperative myocardial injury [18]. Existing data suggested that NLR could be used as a marker for risk assessment in patients with acute [19] and chronic [20] heart failure, and was closely associated with increased all-cause mortality [21]. Recently, many studies emphasized that inflammation was involved in the formation of pulmonary hypertension. In children with CHD, especially with continuous left-to-right shunt, pulmonary blood flow and pressure increase, resulting in pulmonary artery endothelial cell dysfunction and pulmonary vascular remodeling. Mechanical stimulation of pulmonary vascular wall by continuous left-to-right shunt lead to inflammatory response of pulmonary vascular wall. Pulmonary vascular endothelial cells over-released vasoconstrictive factors such as endothelin, causing pulmonary vasoconstriction, thus resulting in increased pulmonary arterial pressure and eventually pulmonary hypertension [22]. In the present study, we firstly applied NLR as a predictive factor in pediatric patients with CHD-PAH undergoing cardiac surgery. Our results indicated that NLR was closely correlated with early post-operative complications of liver and kidney function as well as poor clinical outcomes.

At present, there are few reports on the relationship between NLR and the prognosis of children with CHD-PAH. Therefore, this study further evaluated the predictive value of NLR for the prolongation of mechanical ventilation time. Prolonged mechanical ventilation time is one of the most important outcome indicators for cardiac surgery. Once it occurs, it may lead to pulmonary edema, atelectasis and ventilator-associated pneumonia, which would further exacerbate the illness state of the children [23]. The longer duration of mechanical ventilation could result in a significant increase in mortality [24]. Faruk et al. reported that NLR was capable of predicting successful extubation following prolonged intubation in patients [25]. In the present research, we discovered that the areas under the curve of 24 h, 48 h and 72 h prolonged mechanical ventilation predicted by NLR before and after operation were statistically significant. Furthermore, it was observed that pre-NLR had high specificity and post-NLR had high sensitivity in predicting prolonged mechanical ventilation, suggesting that monitoring the level of NLR in children with CHD-PAH was helpful to perceive the occurrence of post-operative adverse events such as prolonged mechanical ventilation. Consequently, more attention should be focused on children with elevated NLR before and early after surgery, and effective interventions should be timely carried out to prevent the occurrence of adverse events. For example, patients in high risk before operation can be treated with oxygen inhalation, improvement of cardiac function and reduction of pulmonary artery pressure by using of targeted drugs. After operation, effective measures for prevention low cardiac output syndrome, acute lung injury and ventilator-associated pneumonia are beneficial for successful weaning. Besides, early respiratory rehabilitation exercise was considered to be advantageous for patients with mechanical ventilation [26] so as to improve the clinical prognosis of children.

There are still several limitations of the present study that may provide further extension of the research. Firstly, the PASP of the children with CHD-PAH was measured by echocardiography, which was not much accurate compared with the right cardiac catheterization. Secondly, this study was designed retrospectively in a single center with a small sample size. Finally, longer follow-up is needed to predict the effect of NLR on the long-term prognosis of patients.

## Conclusion

In conclusion, pre-operative and post-operative NLR in children with CHD-PAH were closely related to early postoperative complications and clinical outcomes and could predict the occurrence of prolonged mechanical ventilation time to some extent. As a new type of inflammatory marker, NLR is simple, cost-effective and easy to be obtained, thus having great advantages while used in community and primary medical institutions. Based on the present study, we suppose that dynamic monitoring of NLR is beneficial for improving the clinical outcomes of children with CHD-PAH after cardiac surgery.

## Supplementary Information

**Additional file 1: Supplementary Table 1**. Detailed diagnosis of included patients.

## Data Availability

The datasets used and/or analysed during the current study are available from the corresponding author on reasonable request.

## Abbreviations

NLR: Neutrophil to lymphocyte ratio
CHD: Congenital heart disease
PAH: Pulmonary arterial hypertension
CPB: Cardiopulmonary bypass
ICU: Intensive care unit
ROC: Receiver operating characteristic
PASP: Pulmonary arterial systolic pressure
ASD: Atrial septal defect
VSD: Ventricular septal defect
PDA: Patent ductus arteriosus
ALT: Glutamic pyruvic transaminase
AST: Glutamic oxaloacetic transaminase
ALB: Albumin
STB: Serum total bilirubin
CR: Creatinine
BUN: Blood urea nitrogen
UA: Uric acid
RACHS-1: Risk adjustment for congenital heart surgery-1
LVEF: Left ventricular ejection fraction
