# Supplementary material for: Predictive role of perioperative neutrophil to lymphocyte ratio in pediatric congenital heart disease associated with pulmonary arterial hypertension
Source: BMC Surg. 2021 Jan 4;21:3. doi: 10.1186/s12893-020-01009-x (PMC7780205; doi:10.1186/s12893-020-01009-x)
Supplement: Supplementary file 1 — Additional file 1: Supplementary Table 1. Detailed diagnosis of included patients. [file 12893_2020_1009_MOESM1_ESM.docx]

**Supplementary Table 1.** Detailed diagnosis of included patients.

| **Simple CHD** | **135** |
| --- | --- |
| ASD | 3 |
| VSD | 66 |
| ASD + VSD | 43 |
| ASD + PDA | 2 |
| VSD + PDA | 10 |
| ASD + VSD + PDA | 11 |
| **Complex CHD** | **55** |
| MVR + ASD | 3 |
| MVR + VSD | 14 |
| MVR + ASD + VSD | 8 |
| MVR + VSD + PDA | 2 |
| MVR + ASD + VSD + PDA | 2 |
| TAPVC | 3 |
| PAPVC | 2 |
| PAVCD | 5 |
| TAVCD | 4 |
| Triatrium | 1 |
| Coarctation of aortic arch + ASD + VSD + PDA | 1 |
| Ebstein anomaly | 1 |
| Double outlet right ventricle + ASD + VSD + PDA | 1 |
| Unroofed coronary sinus + ASD | 1 |
| Common truncus arteriosus (Type II) + VSD | 1 |
| Aortic valve prolapse + VSD | 1 |
| Interrupted aortic arch + VSD + PDA | 1 |
| Double-chambered right ventricle + VSD | 1 |
| Double outlet right ventricle + VSD | 1 |
| Triatrium + MVR | 1 |
| Left coronary artery-right atrial fistula | 1 |

CHD: congenital heart disease; ASD: atrial septal defect; VSD: ventricular septal defect; PDA: Patent Ductus arteriosus; MVR: mitral valve regurgitation; TAPVC: total anomalous pulmonary venous connection; PAPVC: partial anomalous pulmonary venous connection; PAVCD: partial atrioventricular septal defect; TAVCD: total atrioventricular septal defect
